# Does the intubation timeline affect the in-hospital mortality of COVID-19 patients? A retrospective cohort study

**DOI:** 10.3389/fmed.2022.1023229

**Published:** 2022-10-06

**Authors:** Shazia Rehman, Muhammad Ali Shahiman, Mundher A. Khaleel, Ondřej Holý

**Affiliations:** ^1^Department of Biomedical Sciences, Pak-Austria Fachhochschule, Institute of Applied Sciences and Technology, Haripur, Pakistan; ^2^Department of Urology, and Renal Transplantation, Benazir Bhutto Hospital, Rawalpindi Medical University, Rawalpindi, Pakistan; ^3^Department of Mathematics, College of Computer Science and Mathematics, Tikrit University, Tikrit, Iraq; ^4^Science and Research Center, Faculty of Health Sciences, Palacký University Olomouc, Olomouc, Czechia

**Keywords:** early intubation, late intubation, COVID-19, acute respiratory failure, emergency unit

## Abstract

**Background:**

Effective strategies for managing coronavirus disease 19 (COVID-19) patients suffering from acute respiratory distress are constantly evolving. The timeline and threshold for transitioning from non-invasive ventilation to intermittent mandatory ventilation in critical cases who develop COVID-19-related respiratory distress are undetermined. The present research intends to investigate if emergency room intubations in COVID-19 patients affect mortality.

**Methods:**

Between January 1, 2021 and June 30, 2021, we retrospectively reviewed chart analysis on all patients with confirmed positive COVID-19 screening and who underwent endotracheal intubation. Depending on when the intubation was performed; early in the emergency room or delayed outside the emergency room, patients were separated into two cohorts. In addition to comorbid clinical manifestations, the quick sequential organ failure assessment (qSOFA) score, and in-hospital mortality were all recorded as demographic and clinical information.

**Results:**

Fifty-eight of the 224 corona-positive patients who underwent intubation had their intubations performed in the emergency room. Age, sex, alcohol use, and smoking status did not significantly differ between the two categories at the baseline. The mean qSOFA score was higher in the early intubation cohort (3.5; *p* < 0.000) along with more underlying comorbidities (3.0; *p* < 0.000). When compared to the late intubation cohort (45.78%), patients treated with early intubation had a significantly greater death rate (67.24%).

**Conclusion:**

In summary, we discovered that patients who underwent intubation in the emergency units exhibited a high quick SOFA score as well as maximum co-morbid conditions than patients intubated somewhere else in the hospital. The findings of our investigation imply that intubating patients too early might be risky.

## Introduction

The coronavirus disease 2019 (COVID-19) pandemic progressed within a few months at the beginning of 2020 from a lone outbreak in the Chinese city of Wuhan to a global disaster of unprecedented scope in the going era (1–4). Without data-driven evidence-based practices, healthcare practitioners were compelled to handle severely infected patients while the novel virus proliferated around the globe. Several early therapeutic procedures were established from prior effective treatments for addressing related pathologies (5, 6). The pathogenesis and administration of severely affected COVID-19 cases are still under investigation. Past evidence from the original coronavirus epidemic in Wuhan revealed that the elderly, patients with co-morbidities, and patients who experienced acute respiratory distress syndrome (ARDS) had a greater fatality risk from COVID-19 pneumonia (7, 8). The timeline and baseline for transitioning from non-invasive ventilation to intermittent mandatory ventilation in critical cases who develop COVID-19-related respiratory distress are undetermined.

Across multiple pandemic epicenters, supply-demand respiratory support may have influenced clinical decision-making considering the use of early vs. delayed or even without intubation (9). In other words, practitioners might be compelled to forgo intubating patients in the context of triage when ventilators are not available. Siempos et al. reported that among 101 patients of laboratory-confirmed COVID-19 patients' early intubation was not associated with worse clinical outcomes compared to delayed or no intubation group of adults (10). In a meta-analysis, it was found no statistically detectable difference in all-cause mortality between patients undergoing early vs. late intubation (11). Studies have shown conflicting results based on treating patients infected with COVID-19 with early or late intubation, though shreds of evidence are still sparse as coronavirus is still active in its variant forms (12–15). The compiled evidence indicates that intubation timings may not influence the COVID-19-related mortality and morbidity in critically ill cases (11). These findings could support a wait-and-see strategy, which could result in fewer intubations. Therefore, it is vital to reconsider appropriate standards and additional epidemiological research in this situation is indeed warranted to support the earlier findings.

Clinicians have a distinct predicament while treating corona-infected patients within the emergency units. The decision of whether to conduct endotracheal intubation or use non-invasive modalities of resuscitation is particularly controversial. An intubated patient's inherent risks of complications like ventilator-associated pneumonia, clinical manifestations, ventilator-induced lung injury, and consequences of extended sedation and incapacitation must be balanced against the immediate need to optimize oxygenation and breathing function (16). It is believed that COVID-19 patients are more likely to experience peri-intubation hypoxemia (below 80 mm Hg), which was previously shown to occur in 10% of intubations (17). By contrasting patients treated with endotracheal intubation for respiratory failure caused by COVID-19, our research seeks to add to these findings. This is, as far as we are aware, the first research to compare the outcomes of corona-infected patients who underwent intubation in both the emergency room and the critical care unit. Contrary to current practice recommendations, which urge early (immediate) endotracheal intubation of severe cases of COVID-19, we postulate that there is no mortality advantage to being intubated urgently in the emergency room over being intubated in critical care units, operating rooms, or another more controlled environment.

## Materials and methods

### Data source and study population

The present investigation was carried out retrospectively based on chart review without any patient involvement. The information was retrieved from nine public hospitals in the district of Rawalpindi, Pakistan. Informed consent was not mandatory for the study due to its nature. The data were fetched from the computerized medical records of patients from the selected hospitals. Participants with a confirmed positive COVID-19 screening test conducted on January 1, 2021, and June 30, 2021, were determined through the computerized medical record. All patients who had experienced endotracheal intubation were also discovered on this list, separated from their own collected data, and also had their intubation spot and time assessed manually by chart review. Epidemiological data along with clinical outcomes were also recorded through chart review. We also documented all patients' related comorbidities (such as hypertension, asthma, chronic kidney disease, dyslipidemia, malignancy, congestive heart failure, coronary artery disease, chronic obstructive pulmonary disease, and immunocompromised status), and we allocated each pre-existing comorbidity (on hospital arrival) 1 point to generate an overall comorbidity score. This enabled us to compare the demographics of the two groups. Adult patients (aged 18 ≥) who underwent endotracheal intubation within the hospital and had a confirmed positive COVID-19 polymerase chain reaction (PCR) screening within the specified research period were eligible for inclusion. Patients who didn't undergo endotracheal intubation and patients with possible corona infected symptoms and yet negative outcomes from screening were excluded from the study sample. In our study, patients who were intubated in an emergency were described as early intubation whereas patients who were intubated in a more contained environment (for instance intensive care unit or operating room) were described as late intubation. Patients were grouped according to intubation disposition and placement as no particular period was defined for early or late intubation.

### Ethical considerations

All protocols were carried out in compliance with the guidelines established by our institution's Ethics Committee (*MH-2021/PK-ISH-1002*) and the Declaration of Helsinki's principles.

### Statistical analysis

With an independent *t*-test or Wilcoxon rank sum test for continuous variables, where appropriate, and χ^2^ test for categorical variables, we compared the outcomes of intubated and non-intubated emergency department patients separately without adjusting for any variables. Using SAS version 9.3 (SAS Institute Inc., Cary, NC, United States), the overall analysis was performed with a significant threshold of < 0.05 and without accounting for multiple comparisons.

## Results

Table 1 summarizes the demographics of COVID-19 patients with early intubation rates of 25.89% and late intubation rates of 74.11%. An aggregate of 224 infected patients who required intubation while they were hospitalized was examined. Of these patients, 58 underwent early intubation at the emergency room, whereas the rest 166 underwent late intubation after receiving intubation elsewhere within the hospital. In general, the baseline demographics of these two groups were comparable. Male patients accounted for a greater proportion of the 224 patients in the emergency room, with 39 experiencing early intubation and 118 enduring late intubations. The mean age of patients with early intubation was estimated to be 71 [60–82] whereas those with late intubation had 67 [56–78]. Overall, approximately 70.69% of the early intubation and 57.83% of the late intubation cohort were identified as smokers. Likewise, 25 patients in the early intubation group and 54 patients who had late intubation were alcohol dependent. Finally, there were comparable pre-existing comorbidities in both cohorts, with averages of 3.5 and 3.0 in the groups receiving early and late intubation, respectively.

**Table 1:** Baseline demographics of COVID-19 patients intubated early and late (*n* = 224).

	**With intubation (early = 58) *n* (%)**	**Without intubation (late = 166)**	***P*-value**
		***n* (%)**	
**Age (years)**			
Mean (range)	71 (60–82)	67 (56–78)	0.22
**Sex**			
Male	39 (67.24)	118 (71.08)	0.67
Female	19 (32.76)	48 (28.92)	
**Smoker**			
Yes	41 (70.69)	96 (57.83)	0.95
No	17 (29.31)	70 (42.17)	
**Alcoholic**			
Yes	25 (43.10)	54 (32.53)	0.31
No	33 (56.90)	112 (67.47)	
**Overall comorbidities**			
Mean (range)	3.5 (0–8)	3.0 (0–6)	0.07

A statistical value *P* < 0.05 is considered significant.

Table 2 provides the statistics of associated mortality and quick sequential organ failure assessment (qSOFA) scores of the COVID-19-infected patients. In comparison to those who experienced early intubation somewhere else in the hospital and subsequently during their stay after departing the emergency room, patients who had an emergency department (early) intubation had a greater death rate while they were hospitalized. In the early intubation cohort, the mortality rate was 67.24%, whereas, in the late intubation cohort, it was 45.78%. This difference is found statistically significant at *p* = 0.01. Moreover, a statistically significant difference (*p* < 0.000) in the baseline qSOFA scores between the two groups existed, with emergency department intubations having a higher mean score of qSOFA. The average qSOFA score for patients in the early intubation group was 3 while the average qSOFA score for patients in the late intubation cohort was found 2.

**Table 2:** Mortality and qSOFA score in COVID-19 patients with early and late intubation (*n* = 224).

	**With intubation (early = 58)**	**Without intubation (late = 166)**	***P*-value**
Deceased	39 (67.24)	76 (45.78)	0.01
Survived	19 (32.76)	90 (54.22)	
**qSOFA**			
Mean (range)	3 (1–3)	2 (0–3)	0.000

qSOFA, quick sequential organ failure assessment.

A statistical value *P* < 0.05 is considered significant.

## Discussion

In contrast to patients who underwent intubation later during their hospital stay, we expected that there was no mortality advantage for cases who exhibited intubation immediately in the emergency room. According to the current study outcomes, patients who had emergency department intubation had an elevated rate of in-hospital mortality than those who underwent intubation later in the admission process. While there were no considerable variations in the baseline demographics of age, sex, smoking, and alcoholic status between the two cohorts of patients, there was a noticeable difference in the total number of comorbidities though not statistically significant. Based on their qSOFA scores, the patients who had early intubation also had greater baseline acuities. In the period leading up to the coronavirus epidemic, it was considered that the optimum outcome for infected patients who presented with hypoxemia was achieved with early intubation.

After several months of managing severe patients of COVID-19, the Chinese Society of Anesthesiology Task Force presented guidelines for intubating patients experiencing respiratory arrest resulting from corona-infection (18). These parameters comprise severely sick patients with persistent tachypnea, hypoxemia, or respiratory distress within 2 h of non-invasive oxygen administration. This guidance was offered because immediate intubation would be physiologically beneficial by preventing a condition referred to as self-induced lung deterioration (19). According to expert opinion articles, increasing respiratory efforts could result in self-induced lung injury. Theoretically, intubation and mechanical ventilation protect against self-induced lung injury by lowering inspiratory effort and tidal volumes (20, 21). Some of the health practitioners in Wuhan who previously treated COVID-19 patients bemoaned the fact that patients acquired extra post-exercise oxygen consumption (EPOC) and were intubated relatively later in the disease progression (22). This resulted in an explicit supposition suggestion that COVID-19 patients be ventilated earlier in the illness course to avert lung impairment. Current evidence on COVID-19 treatment and consequences, however, has thrown this paradigm into doubt (12, 13, 21, 23–25). COVID-19 has different pathophysiology than more conventional ARDS and is more prone to non–invasive methods of ventilation such as high-flow nasal cannula (26). Therefore, the emphasis of initial care for COVID-19-induced hypoxemia is on non-invasive methods of oxygenation (27). Since there is insufficient clinical evidence to support immediate intubation in infected patients, some experts advise against doing so. They also point out that the hypothesized theory of corona-infected patient-induced lung injury is relatively speculative (16, 28). Although there are limited investigations in the literature on when to intubate COVID-19 patients, modest studies have shown inconsistent fatality rates in patients intubated sooner in their course of illness (29–31). Non–invasive ventilation has not been advocated for in the literature since it could aerosolize COVID-19 spores (32). Some medical professionals working in the emergency department probably chose to do intubations instead of non–invasive oxygenation for this rationale.

The outcomes of our research lead us to conclude that delayed intubation in COVID-19 patients is warranted. The intubation time has been the subject of inconsistent evidence, which has improved gradually as additional research has come to the fore. In our research, early intubation was performed on severely ill patients as demonstrated by high qSOFA scores; nevertheless, this resulted in raised mortality rate. This is probably the consequence of steadily increasing lung injury driven by mechanical ventilation. The quick SOFA score is a bedside prompt that may identify patients with suspected infection who are at greater risk for a poor outcome outside the intensive care unit. This is probably owing to the enhanced lung injury brought on by mechanical respiration. Therefore, we anticipate that additional investigation may identify the factors that render other COVID-19 treatment options in severe cases preferable to early intubation in terms of reducing mortality. These might involve proning, non–invasive ventilation, a high-flow nasal cannula, and some additional medicinal therapies. As previously demonstrated that early intubation is causing severe outcomes leading to increased lung injury at an early stage of disease progression, which results in aggravating hypoxemia and exacerbated multi-organ dysfunction, ultimately elevating mortality (33). The results of our findings validate this assumption. The existing literature highlights the discrepancies between lung injury due to COVID-19 infection and ARDS resulting from multiple etiological factors as pathophysiological understanding related to COVID-19 cases has progressed (34, 35). Reduced lung compatibility is a characteristic of common ARDS, and it is treated using lung-protective ventilation techniques (36). Unfortunately, mechanical ventilation is attributed to significant mortality in COVID-19 patients and may exacerbate acute respiratory difficulties (37–39).

The contemporary COVID-19 therapies focused on prolonging endotracheal intubation and using non–invasive modalities of resuscitation for avoiding respiratory failure (40, 41). These modalities involve self-proning techniques, continuous positive airway pressure or bilevel positive airway pressure, and high-flow or mid-flow nasal cannulas (42). Endotracheal intubation is presently considered the ultimate option for refractory hypoxia. According to health practitioners, lower oxygen saturations are acceptable as long as patients don't develop symptoms of altered mental state or respiratory distress. The evidence provided in the present work adds to the growing body of research that suggests infected patients with COVID-19 should undergo delayed intubation.

Healthcare professionals administering COVID-19 patients find it challenging to handle cases rapidly in the event of a significant deterioration due to the requirement for compliance with airborne precautions and personal safety equipment (43). If respiratory function deteriorates since emergent intubation may raise the risk of nosocomial infection for healthcare professionals, thus treatment recommendations advise timely intubation in a supervised environment (41). Our findings should thus be rigorously implemented in medical practice, and a prediction model that can spot COVID-19 patients who are critically ill and at risk for respiratory distress that necessitates intubation is required. To adequately identify individuals who need proactive resuscitation, additional investigation on this subject with increased sample size is thus recommended.

There are a few limitations to this research. First, clinicians were free to choose whether to conduct endotracheal intubation or administer non–invasive oxygenation at their convenience. The possibility of a selection bias is the second drawback. Patients who had intubation early in their hospital stay were probably worse when they were admitted, therefore increased mortality was anticipated. This is evident by the fact that the early intubation cohort in this research had a higher quick SOFA score than the late intubation group. Additionally, since septic shock manifests at a later stage of the illness, the quick SOFA score has been reported to be of limited use in the assessment of patients infected with COVID-19. We had the opportunity of incorporating other conventional COVID-specific prognosis metrics, however, they were established after our investigation was performed. We additionally considered performing a Propensity Score Matched (PSM) method in our investigation to determine whether the variation in fatality persisted despite the variation in the quick SOFA score, but the selected sample size was relatively insufficient to do so. Additional constraints of our research also include its retrospective aspect and insufficient sample size. A significant prospective experiment contrasting early and late intubation might be advantageous in the future. Inadequacies highlighted earlier; these findings contribute to the growing body of research that indicates that early intubation of corona-infected patients had no mortality improvement.

In summary, we discovered that infected patients of COVID-19 who were intubated in the emergency units exhibited a high quick SOFA score as well as maximum co-morbid conditions than patients intubated somewhere else in the hospital after assessing 224 study participants. The findings of our investigation imply that intubating patients too early might be risky.

## Data availability statement

The data analyzed in this study is subject to the following licenses/restrictions. Data available on request due to restrictions of privacy implemented by the institution. The data presented in this study are available on request from the corresponding author. The data are not publicly available due to institutional restrictions. Requests to access these datasets should be directed to ondrej.holy@upol.cz.

## Ethics statement

The study was conducted in accordance with the Declaration of Helsinki and approved by the Institutional Review Board of the Ministry of Health, Pakistan (Reference: MH-2021/PK-ISH-1002). Written informed consent for participation was not required for this study in accordance with the national legislation and the institutional requirements.

## Author contributions

All authors listed have made a substantial, direct, and intellectual contribution to the work and approved it for publication.

## Funding

The work was supported by the Faculty of Health Sciences, Palacký University, Olomouc (Grant No. IGS_FZV_22003).

## Conflict of interest

The authors declare that the research was conducted in the absence of any commercial or financial relationships that could be construed as a potential conflict of interest.

## Publisher's note

All claims expressed in this article are solely those of the authors and do not necessarily represent those of their affiliated organizations, or those of the publisher, the editors and the reviewers. Any product that may be evaluated in this article, or claim that may be made by its manufacturer, is not guaranteed or endorsed by the publisher.
